# A phylogenomic perspective reveals mitochondrial-nuclear discordance and previously undescribed species nested within a widespread East African Reed frog species (*Hyperolius substriatus* Ahl, 1931)

**DOI:** 10.1371/journal.pone.0318951

**Published:** 2025-04-16

**Authors:** Lucinda P. Lawson, Gabriela B. Bittencourt-Silva, Werner Conradie, Daniel M. Portik, Simon P. Loader

**Affiliations:** 1 Department of Biological Sciences, University of Cincinnati, Cincinnati, Ohio, United States of America; 2 Department of Zoology, Field Museum, Chicago, Illinois, United States of America; 3 Herpetology, Natural History Museum, London, United Kingdom; 4 Port Elizabeth Museum, Beach Road, Humewood, Port Elizabeth, South Africa; 5 Department of Nature Conservation Management, Natural Resource Science and Management Cluster, Faculty of Science, George Campus, Nelson Mandela University, George, South Africa; 6 California Academy of Sciences, San Francisco, California, United States of America; Guangxi University, CHINA

## Abstract

The sub-montane East African Reed Frog, *Hyperolius substriatus* Ahl, 1931 (Spotted Reed Frog) has a fragmented highland distribution throughout East Africa. Previous studies show extensive mitochondrial divergence between four lineages of African Spotted Reed Frogs that roughly correspond to previously-recognized subspecies. These may have conservation implications if formally described. However, as mitochondrial-based population models only track maternal patterns, further genomic datasets are necessary to assess the distinctness of these lineages in relation to historically recognized morphological subspecies. In this study, we expanded sampling to newly discovered localities and assessed mitochondrial and genomic data to better understand phylogeography and landscape genomics of this species. We found that genomic clades (biparentally inherited) confirm some of the mitochondrial structure (female inherited), but also revealed multiple cases of mitonuclear discordance particularly within the Udzungwa Mountain block, which may have two separate founding events based on peripatric mitochondrial lineages and panmictic genomic signals. Taken together, the three clades within the geographical range of *H. substriatus* through Tanzania, Malawi, and Mozambique correspond to three previously-identified subspecies and lineages, and have both spatially cohesive and population-specific patterns of geneflow and isolation with neighboring highland locations.

## Introduction

Tropical montane amphibians are collectively some of the least understood and most threatened animals on Earth [1–7]. In tropical montane areas, species confined to single mountain blocks or even single valley micro-endemics are common [8–16]. These endemic species are of high conservation value [17,18], but offer limited insights into biogeographical history of a landscape. Those species with distributions spanning multiple mountains offer opportunities to study the speciation process. In particular, in poor dispersers, such as frogs, species distributions formed from allopatric processes show how populations differentiate over time. This necessitates careful consideration of the impact of philopatry, and geographical barriers to understand evolutionary patterns. These evolutionary patterns are important considerations for understanding species units and their conservation [19–21].

**Fig 1:**
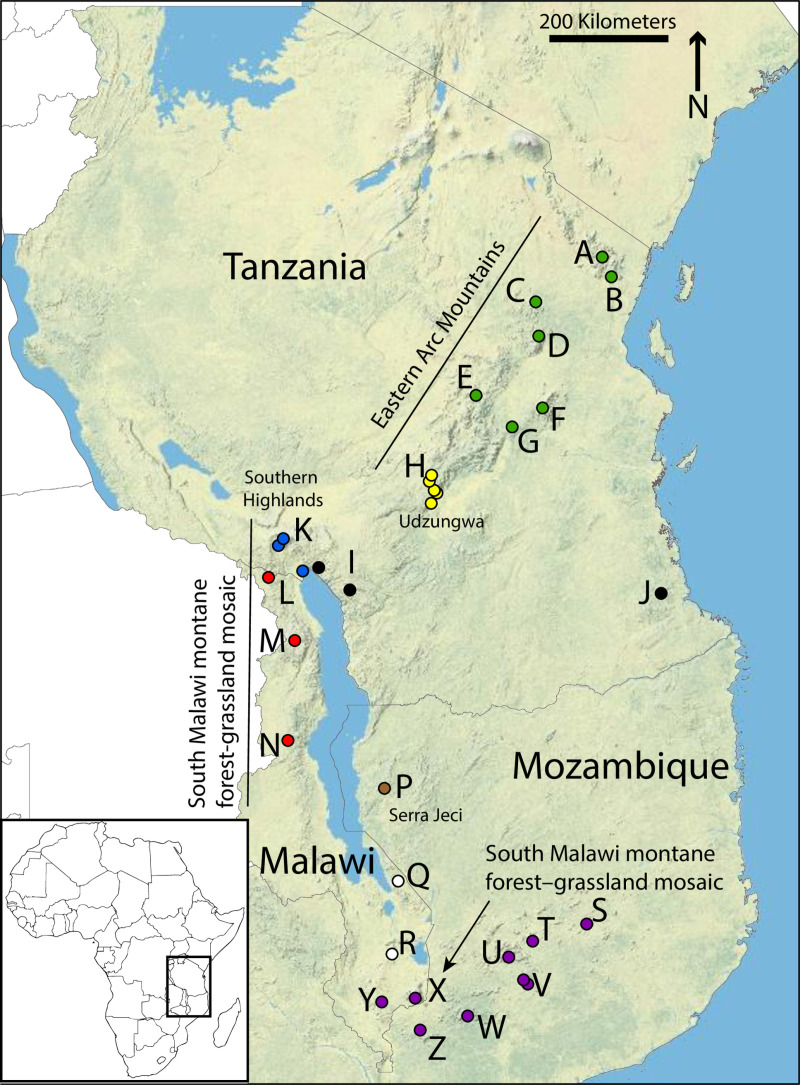
Map of sampled locations in this study. All localities included in this study are indicated. Terrestrial Ecoregions from the World Wildlife Fund are labeled (Eastern Arc Mountains, South Malawi montane forest–grassland mosaic, Southern Rift montane forest–grassland mosaic). Colors match genomic population clusters. Green = northern Tanzanian populations from the northern Eastern Arc Mountains; Yellow = Udzungwa Mountains population from the southern Eastern Arc Mountains. Black = Livingstone Mountains populations and a coastal Tanzanian locality; Blue = populations in the Southern Highlands; Red = northern Malawian populations; Brown = populations in the Serra Jeci Mountains; White = populations in central Malawi; Purple = populations in southern Malawi and Mozambique. Localities: A. West Usambara Mts, B. East Usambara Mts, C. Nguu Mts, D. Nguru Mts, E. Rubeho, F. Uluguru Mts, G. Malundwe Peak, H. Udzungwa Mts, I. Livingstone Mountains, J. Coastal (Lindi Plateau), K. Southern Highlands, L. Misuku Hills, M. Nyika Plateau, N. Vipya Mts, P. Serra Jeci Mts (Njesi Highlands), Q. Namizimu Forest, R. Zomba Plateau, S. Mt Ribaue, T. Mt Inago, U. Mt Namuli, V. Mt Sokon, W. Mt. Mabu, X. Mt. Mulanje, Y. Cholo Mt, Z. Mt. Chiperone. Map created in ESRI with relief basemap (Copyright:© 2009 ESRI).

The Eastern Afromontane Biodiversity Hotspot in East Africa is home to numerous species with extremely limited ranges [19,20,22–27]. Amphibians within this hotspot range from the Kihansi Spray Toad (*Nectophrynoides asperginis*) known from a single valley and categorized as Extinct in the Wild by the IUCN to the widespread *Arthroleptis xenodactyloides* [28]. The Spotted Reed Frog, *Hyperolius substriatus*, is known from submontane regions (and one lowland location) throughout Tanzania, Malawi, and Mozambique (Fig 1), with deep mitochondrial differences between geographic regions (>4%) [29–31]. These deep mitochondrial differences are in line with the suggested range of 2–5% divergences in 16S rRNA between populations to suggest potential cryptic species in amphibians [32,33]. However, before describing new species or subspecies, additional nuclear data are necessary, as maternally-inherited mitochondrial lineages may incorrectly estimate phylogenetic taxonomic units if females have different dispersal patterns than males [21,34–39].

In this study, we investigate the hypothesis that distinct mitochondrially-informed lineages (Sanger-sequenced data of mitochondrial loci and few nuclear markers) first identified in a previous study [29] might be “true” lineages that represent new species. Four lineages based primarily on mitochondrial data and historical species descriptions were proposed: *H. substriatus* in northern and central Tanzania (Ahl, 1931), *H. puncticulatus choloensis* (Loveridge, 1953) in southern Malawi, an undescribed lineage in northern Malawi, and an unnamed lineage from the Udzungwa Mountains in Tanzania (from which we lacked mitochondrial data from many sites) [30,40–42]). The first step is building on the existing dataset with newly sampled geographic populations and expanded mitochondrial and nuclear (Sanger) sequencing. Further, by generating a genomic dataset, we could determine if the mitonuclear discordance seen in Sanger-sequenced loci might be due to incomplete lineage sorting or if they represent true features of the population structure (i.e., maternally-inherited mitochondrial DNA reveals female spatial patterns while genomic DNA shows population connectivity and possibly male movement patterns). To address the additional hypothesis that landscape may contribute to either the mitochondrial (female inherited) or nuclear (biparentally inherited) phylogeographic patterns, we contrasted mitochondrial and genomic clade membership. We discuss our results in the context of major hydrological basins/drainages that may impact migration and gene flow in this montane landscape. In doing so, we created a never-before seen picture of isolation and gene flow within the spatially fragmented Eastern Afromontane Biodiversity Hotspot.

## Materials and methods

### Study permissions and specimen collection

Specimens from Mozambique were collected under the regulations of a research permit administered by the Universidade Eduardo Mondlane Natural History Museum of Maputo (No. 04/2011 and 05/2011) and a credential administered by the Mozambique Ministry of Agriculture. Tanzanian research permits were issued through COSTECH and Tanzanian Wildlife and Forestry departments (2006 and 2007–72-Na-2006–19, 2013–341-NA-2013–121). Malawian research permits (HERP/110/267) were obtained with assistance from the Museums of Malawi.

Populations included in this study span all known populations of *Hyperolius substriatus* from northern Tanzania to southern Malawi and adjacent Mozambique (Fig 1; colors represent genomic-identified clades outlined in the results section).

All specimens for this study were collected in accordance with animal ethics guidelines established in the institutions of authors including the University of Cincinnati (IACUC 21-04-21-02 (LPL) and the Natural History Museum London, and approved by the Tanzania Wildlife Research Institute (TAWIRI). Specimens were collected by hand at night, and were euthanized using an overdose of benzocaine. This method was used according to IACUC recommendations as it minimizes suffering. Samples of muscle and/or liver were taken from representative individuals and preserved in 95% ethanol. See previous studies [29,30] for further details.

### Genetic sampling

Most Sanger-sequenced loci are included from previous studies with additional sequencing of populations using the 16S rRNA locus to complement the existing dataset and to investigate suspected population breaks (e.g., Udzungwa Mountain populations). In total, 28 new Sanger sequences were added to the dataset of [29]. See Supplementary S1 Table for the full list of samples used with accession numbers and new sampling noted. See Table 1 for number of individuals included in each dataset. Total DNA was extracted from tissue preserved in 95% EtOH from *Hyperolius substriatus* from throughout the known range in East Africa (Fig 1) using the DNeasy blood and tissue kit (Qiagen, Valencia, CA). A total of 199 *H. substriatus* individuals and one outgroup (*H. mitchelli*) were included across populations (S1 Table). Extraction, amplification and sequencing followed standard protocols for Sanger sequencing [24]. Datasets from previous studies [29,30] were combined with new sequencing to create as complete of datasets as possible across mitochondrial and nuclear loci spanning all of the four potential subspecies/mitochondrial groups identified in Bittencourt-Silva et al., (2017). The largest dataset was constructed with the mitochondrial ND2 locus (NADH dehydrogenase exon 2: 1152 bp), with smaller representative samples from mitochondrial 16S rRNA locus (483 bp). Of the mitochondrial datasets, the ND2 dataset has more individuals and encompasses extensive sampling within mountain blocks used to highlight sub-structuring. However, it lacks sampling from two regions which the 16S dataset includes: the Livingstone Mountains and the Southern Highlands of Tanzania. Two additional nuclear genes were also sequenced to compliment the mitochondrial dataset and to match existing genetic datasets of this clade [31]: POMC (Pro-opiomelanocortin: exon 629 bp) and c-Myc (Cellular myelocytomatosis proto-oncogene: exon 2, intron 2, exon 3. 1103 bp). Primers and PCR conditions for ND2, POMC, and c-Myc are the same as in Lawson et al. (2015). Primers for 16S were 16SC and 16SD [43]. Amplification followed standard PCR conditions [44] with the following thermal cycle profile: 2 min at 94 °C, followed by 35 cycles of 94 °C for 30 sec, 46 °C for 30 sec, 72 °C for 60 sec and a final extension phase at 72 °C for 7 min. All amplified PCR products were verified using electrophoresis on a 1.0% agarose gel stained with SYBR Safe DNA gel stain (Invitrogen Corporation, Carlsbad, CA, USA). PCR products were purified using the Qiagen DNeasy DNA Purification System following the manufacturer’s recommendations. DNA sequences were obtained on an ABI PRISM 3730xl DNA sequencer. Editing and assembly of contigs were completed in Geneious Prime 2019.2.1 (https://www.geneious.com). The resulting sequences were aligned in MUSCLE v3.7 [45] in the CIPRES gateway server [46] with default settings. S1 Table provides details on voucher specimens used in this study, their origin and associated GenBank numbers where applicable.

**Table 1:** Sample sizes for all groups (number of individuals).

Genomic Clade	Genetic Dataset (ND2/16S/ POMC/CMYC)	Genomic Dataset	Mountain Blocks
Green	68/6/71/63	3	E. Usambara, W. Usambara, Nguu, Nguru, Uluguru, Rubeho, Malundwe
Red	51/3/51/51	7	Nyika, Misuku, Vipya
Yellow	16/23/17/17	4	Udzungwa
Purple	14/6/13/13	4	Mulanje, Chiperone, Namuli, Socone, Cholo, Inago, Mabu, Ribaue, Lico
White	9/2/9/9	3	Zomba, Namizimu
Brown	3/2/2/0	3	Serra Jeci
Black	2/5/2/2	3	Livingstone, Coastal Forests
Blue	0/4/1/1	2	Southern Highlands

Phylogenetic relationships and structure were assessed as haplotype networks for all Sanger sequenced loci (mitochondrial and nuclear) to more clearly show groupings in a landscape population genetics framework and better portray population characteristics that may violate the assumptions of a bifurcating phylogenetic tree. These networks were visualized as unrooted TCS allele networks [47] using PopART v1 [48]. See S1 Table for the dataset used. Pairwise inter-population divergence were calculated using MEGA v7 [49]. Intra-population average distance (p) used uniform rates, pairwise deletion for missing data, and 500 bootstraps.

In addition to haplotype networks, the ND2 dataset, which is the largest, was also assessed as a phylogenetic tree. Bayesian inference (BEAST2 v2.5.2; [50]) was used for these analyses. In order to determine if an outgroup or midbranch rooting would change the phylogenetic relationships, analyses were performed both with an outgroup (*H. mitchelli* – sister lineage to *H. substriatus*; [51] and without (midbranch rooting in BEAST). The topology did not change between these methods. The substitution model used was HKY+I+G, selected using jModelTest2 [52,53] on the CIPRES server, based on Akaike Information Criterion (top model in both AIC and AICc) [54]. A relaxed lognormal clock was used with a Coalescent Constant Population tree prior. The run consisted of 15 million generations logging every 1000 with the first 10% discarded as burn-in. An assessment of model performance was completed in Tracer v1.7.1 [55] for BEAST2, with ESS values above 200 and visual inspection of mixing. A final tree was constructed using TreeAnnotator v 2.6.0 in BEAST2 and viewed in FigTree v1.4.0 (http://tree.bio.ed.ac.uk/software/figtree/).

### Genomic sampling

A smaller dataset (29 individuals), sampling all lineages identified as distinct mitochondrial clades (above) and multiple allopatric populations within clades (e.g., Uluguru, East Usambara, and West Usambara Mountains for the northern Tanzanian clade), was used to create a genomic single nucleotide polymorphism (SNP) dataset through double-digest RAD-sequencing (ddRAD-seq) (Table 1). As this dataset was initially created to determine if the previous pattern of mitonuclear discordance was due to incomplete lineage sorting of Sanger-sequenced nuclear loci, it has representatives from all clades identified in either the mitochondrial or nuclear Sanger datasets but is smaller than these datasets. A larger dataset of ~50 samples were initially intended to be included, but as many samples were collected 20+ years earlier, many did not pass quality control for new genomic analyses. See Table 1 for sample sizes. Samples were extracted from fresh tissues (DNEasy), quantified using a Qubit 2.0 DNA HS Assay (ThermoFisher, Massachusetts, USA), and normalized to 500 ng DNA when possible (>100 ng minimum). These were then sent to Admera Health Genomics (Genohub project, USA) for sequencing. Quality was assessed by Tapestation genomic DNA Assay (Agilent Technologies, California, USA). Adapters were annealed based on the enzymes EcoRI-MspI. Double digestion was performed based on the restriction enzymes. The samples were pooled before size selection (250–350 bp) and enrichment of target DNA fragments was performed to prepare libraries by PCR amplification. Final libraries quantity was assessed by Qubit 2.0 (ThermoFisher, Massachusetts, USA) and quality was assessed by TapeStation D1000 ScreenTape (Agilent Technologies Inc., California, USA). Illumina® 8-nt dual-indices were used. Equimolar pooling of libraries was performed based on QC values and sequenced on an Illumina® Novaseq platform (Illumina, California, USA) with a read length configuration of 2x150 for 0.8M PE reads per sample (0.4M in each direction).

The STACKS pipeline (v2.60) was used to identify SNPs [56–58] using --paired flags to incorporate paired-end sequencing. STACKS parameters were explored through the R80 method [59] and estimates comparing values 2–12 were explored for M=n for de novo ddRAD-seq assembly and SNP discovery, as well as downstream analyses. As only 1–2 individuals were representative of each population, all individuals were treated as a single population for the R80 method (STACKS author’s recommendation, J. Catchen). M & n = 10 were ultimately used to balance the diversity of samples within the dataset and identifying true SNPs. The m parameter (minimum number of reads to seed a stack) was left at the default value of 3. In total, 967,838 loci were generated. After filtering, 24,566 loci were retained, composed of 52,5356 variant sites. The effective per-sample coverage had a mean of 14.9x (stdev=2.2x, min=9.5x, max=20.2x) with a mean length per locus of 259.5. This dataset was then filtered to only contain loci present in all populations, yielding 9,862 loci with 51,857 variant sites. The mean length of loci was 280.30 bp. Finally, only the first SNP of each locus was included in the final dataset to create unlinked loci, crucial for SNAPP and STRUCTURE analyses in this non-referenced dataset. This “first SNP” bi-allelic dataset of 8,837 SNPs (reduced from 9,862 to remove SNPs which had “N” in the first SNP position for any population) was used in all further phylogenetic analyses.

Three genomic clustering methods were explored. First, STRUCTURE v2.3.4 [60] was used for population genetic analyses. The value of lambda was investigated to determine if this SNP dataset deviated from lambda = 1, as recommended in the STRUCTURE manual. K=1 was run with 10 iterations estimating lambda. As lambda was always approximately 1 (~1.1 average), all analyses were conducted with the default lambda value of 1. Population clusters (K) between 1–12 were assessed, with 10 iterations each. The burnin period was 100,000. The length of the MCMC was set at 200,000 for each run. We used the admixture model without a population prior. All other parameters were set to default values. The program STRUCTURESelector [61] was used to estimated population clusters. This method implements medmedk, medmeak, maxmedk and maxmeak statistics to better estimate clusters with uneven population sampling (as this dataset contains) [62]. Two further clustering methods were also conducted: a non-Bayesian Discriminant Analysis of Principal Component (DAPC) clustering based on PCA and a Discriminant Analysis using the *glPca* and *dapc* commands implemented in the *adegenet* R package [63].

In creating phylogenetic trees, two methods were employed to better capture potential variation in phylogenetic relationships: A phylogenetic tree was created through a SNAPP analysis in BEAST2 (SNP and AFLP Package for Phylogenetic analysis, [64]), and a Genetic Distance Tree was created with the R packages *vcfr*, *poppr*, and *ape* [65–67]. To create a Genetic Distance Tree, the UPGMA algorithm was used with 100 bootstrap replicates to assess branch support (midpoint rooting of the longest branch, as inherent to UPGMA).

In a SNAPP analysis [68], bi-allelic SNPs were used to derive a posterior distribution of putative species trees through estimating the probability of allele frequency changes across nodes given the data. The coalescent-based species tree was inferred with the following model parameters deviating from default settings: unselecting “Include non-polymorphic sites” and selecting “Use Log Likelihood Correction” in Model Parameter. The SNAPP analysis was run for 100,000,000 iterations, with 10% burn-in, and sampling every 1,000 iterations. To ensure that the effective sample sizes (ESS) across parameters were ≥200, all results were assessed in Tracer. The SNAPP tree was viewed in Densitree v2.2.7 [69]. A range of models and prior values were evaluated to assess the robustness of the generated trees. The program was run both by treating each sample as an individual species or population, and by assigning them to the clades identified in the “individual” analyses and the structure analysis. As results were largely consistent (see below, variation in placement of the Southern Highlands population), parameters matched recommendations from [70]: mutation rates u and v = 1 with sampling disabled, Coalescent rate = 10, lambda alpha = 2, lambda beta = 200, snapprior alpha = 1, snapprior beta = 250, snapprior kappa = 1, snaprior lambda = 10, rateprior = gamma.

To determine confidence in nodes for the SNAPP tree and the Genetic Distance Tree, posterior probabilities (SNAPP) were calculated with TreeAnnotator in the BEAST package, and bootstrap values (Genetic Distance) were calculated with the poppr function.

We used TreeMix v.1.13 [71] to evaluate migration/introgression among populations identified in STRUCTURE within a phylogenetic framework. We used unrooted trees with default parameters and migration events ranging from 0–8. SNPs were bootstrapped in blocks of 500 for 100 replicates.

### Spatial structure and hydrobasins

Hydrobasins (HydroBASINS) are watershed boundaries and sub-basin delineations derived from HydroSHEDS data at 15 second resolution [72]. Based on earlier studies of *H. substriatus* [30], we mapped the distribution of sampling localities compared to major and minor hydrobasins for a qualitative assessment if either mitochondrial (matrilineal lineages) or genomic (male or both sexes) breaks between groupings corresponded to occurrence in different hydrobasins despite spatial proximity.

## Results

### Genetic results

**Fig 2:**
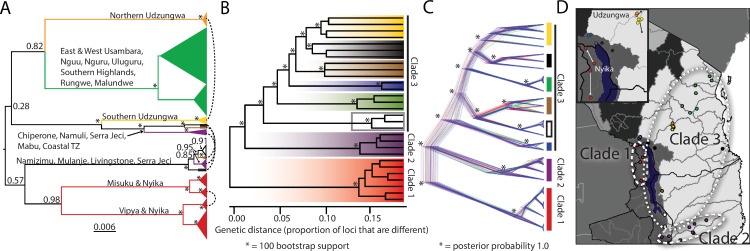
Phylogeographic clades of mitochondrial and genomic loci. A. BEAST2 Bayesian Inference phylogenetic tree of mitochondrial ND2 dataset. Posterior probabilities shown at nodes with branch lengths proportional to nucleotide divergence (asterixis indicated 1.0 posterior values). Names match the locations indicated by letters in Fig 1 and can be referenced from the figure legend. Colors match localities in Fig 1 (based on genomic results) except in the Udzungwa mountain block (yellow in map figure) which are shown in orange and yellow to represent the two unconnected mitochondrial clades (northern Udzungwa in orange and southern Udzungwa in yellow). Mitochondrial and nuclear haplotype networks (ND2, 16S, POMC, and c-Myc) are in supplementary materials. Arcs connecting clades indicate relationships supported by nuclear loci (c-Myc and POMC) that are in discordance with the ND2 mitochondrial tree (S1 Fig). B. Distance tree (proportion of loci that are different) based on genomic dataset. All nodes show 100 bootstrap support, with asterisks displayed on major nodes to denote this. C. SNAPP tree of SNPs. The figure shows the posterior distribution of species tree topologies based on the multi-species coalescent approach with the most likely tree shown in blue and alternate topologies shown in green and red. All major nodes have 1.0 posterior probability, indicated by asterisks. D. Hydrological drainage basins (hydrobasins) and major phylogenetic clades are shown. The three main phylogenetic clusters in the genomic dataset are identified as Clades 1–3. Major hydrobasins were obtained from www.hydrosheds.org, downloaded on February 2011 © HydroSHEDS. Inset: A closer map of the region in south-central Tanzania and norther Malawi is shown encompassing populations that have unexpected mitochondrial breaks given the close spatial proximity. The two mitochondrial haplotypes in the Udzungwa Mountains (yellow and orange) are in two different minor hydrobasins. Likewise, the two central red populations on the north and south slopes of the Nyika Plateau cluster with the other sampling locations of each shared minor hydrobasin. Arrows pointing to the spatial location of the nearest mitochondrial connection for these four localities are shown.

Mitochondrial analyses show significant population structuring, sometimes even within mountain blocks for mitochondrial data (e.g., Udzungwa Mountains, shown as yellow and orange circles in Fig 2A and S1 Fig). Both mitochondrial genes (ND2 and 16S) showed similar patterns (ND2: Fig 2A and S1 Fig; 16S: S1 Fig). While some mountain blocks were monophyletic for all populations within (e.g., each mountain block within the northern section of the Eastern Arc Mountains (green), Fig 2A and S1 Fig), others showed unexpected spatial relationships in the mitochondrial dataset which were supported in both the 16S and ND2 analyses (Fig 2A and S1 Fig). For example, three individuals were collected in one population at Serra Jeci (also known as Njesi Highlands, Mozambique) (Fig 1, brown), however two clustered with one clade of the mitochondrial phylogenetic tree, and the other clustered with an entirely different clade (brown Fig 2A and S1 Fig). Additionally, populations within the Udzungwa Mountain block (yellow) from the southern Eastern Arc Mountains fell into two distantly related clusters: “northern Udzungwa Mountains” (yellow, Fig 2A and S1 Fig) and “southern Udzungwa Mountains” (orange, Fig 2A and S1 Fig). All cases of population relationships which violated mountain block monophyly (which also corresponded to deviations from genomic clustering) are shown with arcs (Fig 2A).

Nuclear loci (POMC and c-Myc) showed many of the same major clusters as the mitochondrial data (e.g., northern Malawi cluster, red), but overall had different structure patterns (S1 Fig). For example, in mitochondrial data, Vipya Highlands and Nyika Plateau 1 clustered apart from Misuku Hills and Nyika Plateau 2 on the other side of the plateau (Fig 2A, arc in red clade between two clusters). No such structuring was present in c-Myc or POMC. Likewise, the northern and southern Udzungwa populations were intermixed or adjoining in nuclear loci, unlike mitochondrial results which resolved them into separate clades. As we could not determine whether these slower evolving loci were not able to capture real population structure due to incomplete lineage sorting or if they really told a different story than the female-inherited mitochondrial markers, subsequent genomic analyses were included samples from each cluster identified in the mitochondrial analyses.

Pairwise population distances of the 16S data ranged from over 5% at the highest (purple clade compared to both the green and blue clades; colors correspond to genomic groupings below) to slightly below 3% (white population compared to brown, red, and yellow). Full table of pairwise distances and error values shown in Supplementary File S2 Table.

### Genomic results

**Fig 3:**
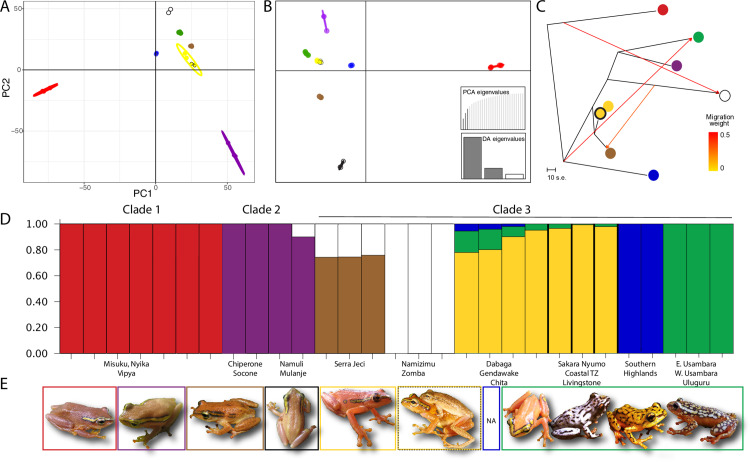
Genomic structuring measures. A. Principal component analysis (PCA) on SNP dataset. Each point represents the SNP profile of an individual. The X axis represents the variance explained by PC1 (29%), and Y axis represents the variance explained by PC2 (16%). Ellipses include 95% of the data for each population. B. DAPC analysis with PCA eigenvalues and DA eigenvalues inset. C. Treemix estimate of unrooted phylogenetic tree with migration. Sizable migration was inferred across many branches of the phylogenetic tree (orange and red arrows). Each population is represented by a colored circle. Coloration of populations matches STRUCTURE plots (the three black localities in Fig 1 are shown as yellow outlined with black as in Fig 2D). D. Genetic structure plot from STRUCTURE (K = 7). Each vertical bar corresponds to one individual. E. Photos of individuals from each population except Southern Highlands. Colors match localities in Fig 1, except the coastal Tanzania and Southern Highlands group (black in Fig 1) are colored as yellow with black outline due to STRUCTURE clustering with Udzungwa Mountain samples (yellow).

All genomic analyses (distance methods, phylogenetic methods, and structuring methods) showed three main patterns. First, there were eight monophyletic population groups (Figs 2B, 2C, 3A, 3B). Two of these groups (localities in Fig 1: the Udzungwa Mountains populations (yellow) and the coastal Tanzania/Livingstone Mountains groups (black)) are members of the same group in STRUCTURE analyses; see below Fig 3D). These groups can be interpreted into three main lineages (clusters from Fig 3A and deepest branches in the phylogenetic trees in Fig 2B and 2C, shown in Fig 2D). Second, these groupings correspond to spatial structuring of mountain blocks, with each mountain or group of mountains containing one monophyletic unit (Figs 2D and 3D). This contrasts with mitochondrial data, which showed multiple distantly related lineages occupying the same mountain blocks in some cases (Figs 2A). Finally, these groups correspond to three major clades: A northern Malawian clade corresponds to a previously identified new lineage [29,30], a southern Malawian and Mozambican clade that corresponds *Hyperolius puncticulatus choloensis* Loveridge, 1953 [40], and a Tanzanian and northern Mozambican clade corresponds to the type locality of *H. substriatus* Ahl, 1931 [41] (Figs 2 and 3).

Distance measures, PCA analyses, and DAPC showed highly structured populations (Figs 2B and 3) with no overlap across distant localities except the Livingstone Mountains of southern Tanzania to Coastal Tanzania, and the Udzungwa Mountains (Fig 1, black and yellow clades, yellow and yellow with black outline points in Figs 3A and 3B). This is corroborated by STRUCTURE analyses (see Fig 3D, yellow blocks and yellow blocks with black borders). In PCA and DAPC analyses (Fig 3A, B), Clade 1 (the northern Malawi clade red) was always the most distant cluster (in line with both phylogenetic trees). In STRUCTURE analyses (Fig 3D), K=6 (medmedK, medmeanK) and K=7 (maxmedK, maxmeanK) were both supported. As K=7 gave results which clearly corresponded to population clusters, we show this result (S2 Fig).

STRUCTURE analyses (Fig 3D) identified possible gene flow (current or ancient) or closely related populations [73] within Clade 3 between the Namizimu and Zomba population (white) and the spatially adjacent Serra Jeci population (brown), and to a lesser extent to Clade 2 (purple, populations in southern Malawi and Mozambique). Additionally, populations in the northern Eastern Arc Mountains (green) and southern Eastern Arc Mountains (yellow) also showed small amounts of gene flow, with the Southern Highlands population (blue) also implicated.

Treemix (Fig 3C) showed significant migration between many populations, in particular between populations that are spatially close to each other but occupy distant phylogenetic relationships in the phylogenetic constructions. The overall phylogenetic tree is similar to the distance tree and genomic trees, with the exception that the southern Malawi/southern Mozambique population (Clade 2, purple) and the Southern Highlands population (blue) have essentially switched positions. At higher values, most migration events were estimated at ~0, but 3 major migration events had strong signal (Fig 3C).

### Spatial structure and hydrobasins

Two major hydrological basins/drainages (hydrobasins) encompass all of the populations of *H. substriatus* that are known (Fig 2D). The northern Malawi population (red, Clade 1) is the only clade deeply within the Malawian hydrobasin, but unrelated populations that are members of other clades (blue, some black populations) were found at the border of this hydrobasin (Fig 2D). Conversely, the light grey Mozambican/Tanzanian hydrobasin contains multiple closely related clades but not others (e.g., the blue and black populations should be within this basin if major hydrobasins dictated clade membership for the three primary clades).

Minor hydrobasins (light grey lines, highlighted in inset figure) exactly straddle some of the unexpected mitochondrial genetic breaks between spatially adjacent populations within a mountain block. The northern Udzungwa clade (orange, Fig 2A and shown on map 2D) and the southern Udzungwa clade (yellow, Fig 2A and shown on map 2D) are on different minor hydrobasins separated by a third in between (inset from Fig 2D, see arrow pointing to spatial location of close mitochondrial connections). Likewise, the deep genetic split within the two populations on the north and south slopes of the Nyika Plateau (Fig 2A (two “Nyika” localities) and inset from Fig 2D (see arrow pointing to spatial location of close mitochondrial connections)) each fall within the same minor hydrobasin as their nearest mitochondrial cluster (northern Nyika population clusters with the Misuku population to the north, and the southern Nyika population clusters with the Vipya populations to the south within the same minor hydrobasin (inset from Fig 2D).

## Discussion

Based on earlier studies of *H. substriatus*, we anticipated that additional sampling localities would help clarify the relationships of the four main clades originally proposed in Bittencourt-Silva et al. (2017). However, additional mitochondrial and nuclear Sanger-sequenced samples instead confirmed mitonuclear discordance between populations and made incomplete lineage sorting of nuclear loci unlikely as multiple mitochondrial markers showed the same relationships to each other, and multiple nuclear markers shared alternate relationships. Genomic data confirmed and further clarified the earlier nuclear dataset, yielding an entirely new perspective on population structuring and conflicting phylogenetic signals linked to differences in movement between the sexes within this spatially fragmented lineage of frogs. While three primary clades were ultimately identified (Fig 2D), they do not match the four mitochondrial clades proposed in Bittencourt-Silva et al. (2017) except for the “northern Malawi” clade (Clade 1) that was also recovered in this study.

The genomic dataset shows seven distinct populations (Fig 3E). Memberships within these populations were consistent and were geographically cohesive across all methods. Additionally, the distance and Bayesian trees in this study recovered the same three primary clades (Fig 2D) but have uncertainty within Clade 3 around the exact relationships between the Serra Jeci population (brown) and the Southern Highlands population (blue) compared to the rest of the members of this clade. Both of these populations had particularly low sample sizes (three and two individuals, respectively) and also have gene flow to other populations indicated by both TreeMix and STRUCTURE analyses. Taken together, these populations might not be easily interpretable within a systematic framework if their spatial locations between the major clades allow low levels of geneflow or complex colonization histories. Additional sampling within these areas (including within the unsampled area in northern Mozambique) and larger datasets from these populations which explicitly address gene flow may help resolve the phylogeographic forces shaping these populations.

Both the Southern Highlands and Sierra Jeci locations also showed mitonuclear discordance. In addition to the genomic indication of introgression with other populations, the Serra Jeci population showed conflicting mitochondrial and nuclear relationships. The three Serra Jeci individuals in our dataset represented two completely different mitochondrial clades within a single locality. The Sanger nuclear dataset as well as the genomic dataset show them as a single population (e.g., STRUCTURE analysis). Mitochondrially, one is a “Namizimu and Zomba” haplotype (white) while the other might represent the “Serra Jeci” haplotype (brown) (Fig 1 for localities). As both mitochondrial and genomic datasets show introgression, the Serra Jeci population appears to have introgression with nearby populations (Namizimu and Zomba) of both males and females (STRUCTURE and TreeMix results). Like Serra Jeci, the population from the Southern Highlands (blue, Fig 1 location K) also shows mitonuclear discordance (S1 Fig, sampling not available for these populations in Fig 2A) with two individuals with a deeply divergent 16S haplotype and one individual with a shared Clade 1 haplotype (shared with an individual from the spatially adjacent Misuku Hills to the south (Fig 1, location L). These relationships are in contrast to the STRUCTURE and TreeMix analyses that predict admixture with Clade 1 populations to the north in the Eastern Arc Mountains, indicating that this population may be difficult to interpret for phylogenetic relationships with the current dataset as Clade 1 and Clade 3 essentially meet at this intersection of major hydrobains and semi-continuous highland habitat (less spatially separated than most of the isolated highland localities of other locations throught the range).

Potential admixture or mitonuclear discordance was also found within Clade 2, as this clade has two mitochondrial clusters (Fig 2A) which each cluster with different populations from Clade 3. One lineage clusters with the Southern Udzungwa Mountains (yellow), Coastal Tanzania (black), and one Serra Jeci individual (brown), and the other clusters with localities from the Livingstone Mountains (black), Namizimu and Zomba (white), and two individuals from Serra Jeci (Figs 2B and 3). Notably, each mountain block is monophyletic in these examples except the Serra Jeci population, as individual populations isolated on mountain blocks or highlands have unique mitochondrial connections.

This pattern is in contrast to results from within the Udzungwa Mountain Range (the southernmost mountain block of the Eastern Arc Mountains; labeled on Fig 1). Within this range, there are two unconnected mitochondrial lineages (shown as orange and yellow in Fig 2A and 2D for mitochondrial data to signify distantly related clades). Populations from the northern part of the Udzungwa Mountains (orange, Fig 2A and 2D, Fig 1 Sanje, Ndundula, and Dabaga) cluster with populations from the rest of the Eastern Arc Mountains to the north (green). While populations in the southern Udzungwa Mountains (Fig 1 Ivalla, Gendawake, Mufindi, Chita, and Mapanda) cluster in the south from Mozambique and coastal Tanzania. Only ~10 km separates the southernmost sampling location of the northern group (Dabaga) from the northernmost location of the southern group (Gendawake), thus the identification of two distantly related mitochondrial clades was unexpected. Mitochondrial data for the northern localities were not included in earlier work [29]. These results were found in both mitochondrial datasets (ND2 and 16S), and both showed consistently different patterns from the nuclear dataset.

Genomic data give an insight into population genetic patterns as the combined Udzungwa population saw introgression from both north and south (Southern Highlands to the south and the northern Tanzanian population to the north). Our results are in line with the hypothesis that the Udzungwa Mountains were colonized by females from two different lineages. Afterwards, females remained philopatric while males dispersed within the Udzungwa Mountain Block, creating one panmictic genomic Udzungwa cluster and connections to the nearby areas (black/yellow cluster in Fig 3D). Though this is untestable without mark-recapture data, further studies should investigate this pattern. Of note, females in the northern Udzungwa Mountains are more pink-hued than females in other Clade 3 populations (pers. obs. and [42], and which might reflect a founding-effect [74]. The contrast between matrilineally inherited patterns and genomic data should be further explored to understand sex-specific founding and migration events within this system [75].

As shown in earlier studies relying more heavily on mitochondrial data [30], drainage basins seem to correspond to the fine-scale phylogenetic structure which deviates from the simpler expectations of Isolation-by-Distance. While this phylogenetic pattern appears true for females, the genomic dataset implies that males move more freely within the landscape (contrast Fig 2A with arcs showing lack of monophyly in mountain blocks to Fig 2B and 2C with 100% spatial monophyly) supporting an Isolation-by-Distance model. Most cases of unexpected genetic breaks correspond to isolation between hydrobasins (inset in Fig 2D); [72].

In this dataset, no populations shared any mitochondrial or nuclear haplotypes and each formed monophyletic clades (except for a shared 16S mitochondrial haplotype between one Southern Highlands individual (blue) and one Misuku Hills individual (red)). Mismatch between mitochondrial and nuclear datasets corresponded to the clustering of these monophyletic groups with each other, implying some differences in initial female founding events and limited current gene flow. Most notably, as mitochondrial structure was much more philopatric than nuclear structure, males and females may move differently through the landscape causing instances of mitonuclear discordance [75,76].

These genomic patterns, suggesting differences between the sexes (e.g., male migration and female philopatry), remain speculative. No direct estimates of migration through mark-recapture or telemetry surveys have ever been completed. As male sex chromosomes are unknown in this lineage, it is not possible to compare directly between mitochondrial and Y-linked genetic regions. Future work with genomic mapping could clarify these issues. Nevertheless, these patterns show that landscape phylogeography in these montane amphibians is nuanced and deserves further study.

In other amphibians, lowland amphibians appear to show a mix of male-biased, female-biased, and non-biased dispersal (summariezed in Helfer et al., 2012). Studies of montane amphibians are still lacking, particularly in populations isolated by large distances across inhospitalble lowlands. For example, a study of salamander populations within close proximity (400m–25km) in the continuous mountains of the Alps found female philopatry and male movement [77], however no between-mountain block analyses were included. Block faulted mountains like the Afromontane Archipelago, where *Hyperolius substriatus* frogs are found, offer a perfect model for comparative phylogeography and landscape analyses as many species have distributions across localities in multiple mountain blocks (e.g., *Hyperolius mitchelli*, *Arthroleptis affinis*, *Leptopelis uluguruensis*). Further studies in montane systems are critical for ecological and evolutionary interpretions, but also conservation planning of dispersal needs for these often overlooked species [78,79].

## Conclusions

Three main lineages exist within the broader *Hyperolius substriatus* species (shown in Fig. 2D). These largely correspond to previously described species/subspecies (*H. substriatus* in Tanzania and northern Mozambique, *H. punctulatus choloensis* in southern Malawi and southern Mozambique, and an identified yet undescribed lineage in northern Malawi). Phylogeographic reconstructions from mitochondrial data did not correctly identify the species tree or major lineages compared to genomic data, raising awareness of the importance for both female and male inherited data when interpreting spatial population structure. In *H. substriatus*, female-inherited mitochondrial data is more phylopatric than genomic data, implying migration within mountain blocks is likely male driven. Future studies explictly quantifying male and female patterns are necessary for a clear understanding of landscape effects on movement and isolation in these montane African frogs.

## Supporting information

S1 Table Table of details on voucher specimens used in this study, their origin and associated GenBank numbers for Sanger sequencing. (XLSX)

S1 Fig Supplementary tree and haplotype figures. Map created in ESRI with relief basemap (Copyright:© 2009 ESRI).
(TIF)

S2 Table Genetic distance results from MEGA. (XLSX)

S2 Fig StructureSelector files. (TIFF)

S1 File Inclusivity in global research. (DOCX)
