# Supplementary figures and images for: A phylogenomic perspective reveals mitochondrial-nuclear discordance and previously undescribed species nested within a widespread East African Reed frog species (Hyperolius substriatus Ahl, 1931)
Source: PLoS One. 2025 Apr 16;20(4):e0318951. doi: 10.1371/journal.pone.0318951 (PMC12002501; doi:10.1371/journal.pone.0318951)

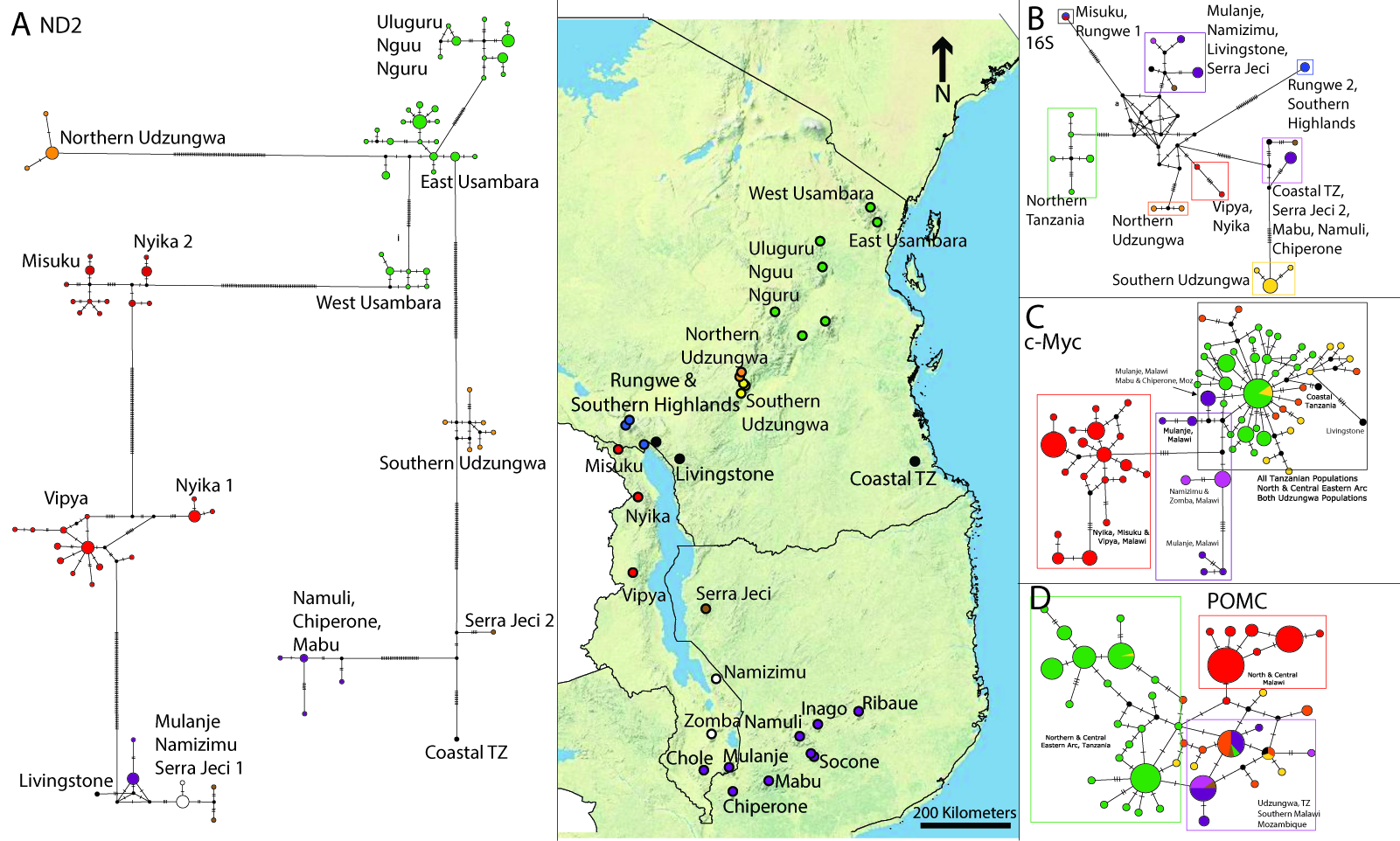

Supplement: S1 Fig — Map created in ESRI with relief basemap (Copyright:© 2009 ESRI). (TIF) [file pone.0318951.s002.tif]

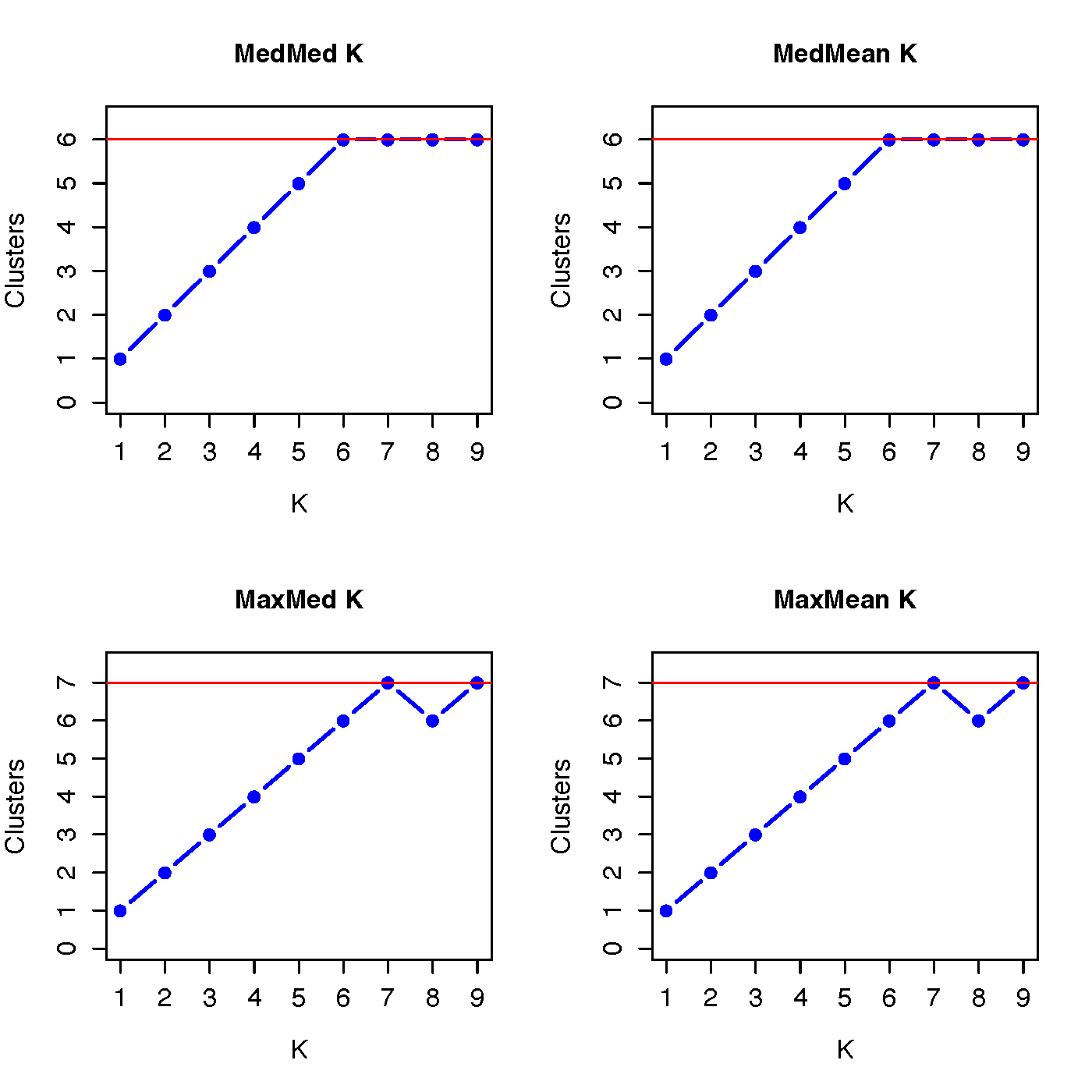

Supplement: S2 Fig — (TIFF) [file pone.0318951.s004.tiff]
